# Psychometric Properties of the Chinese Version of Anxiety Sensitivity Index-3 in Women Diagnosed With Breast Cancer

**DOI:** 10.3389/fpsyg.2020.00012

**Published:** 2020-02-07

**Authors:** Yan Han, Jiang Zhu, Lingyan Li, Huan Zhou, Shichen Li, Jinqiang Zhang, Jie Fan, Yanjie Yang, Xingwei Luo, Xiongzhao Zhu

**Affiliations:** ^1^Medical Psychological Center, Second Xiangya Hospital, Central South University, Changsha, China; ^2^Medical Psychological Institute of Central South University, Changsha, China; ^3^School of Nursing, Nanchang University, Nanchang, China; ^4^School of Public Health, Harbin Medical University, Harbin, China

**Keywords:** anxiety sensitivity, Anxiety Sensitivity Index-3, breast cancer, psychometric properties, measurement invariance

## Abstract

**Background:**

Anxiety sensitivity (AS) is a trait-like predisposing factor for the prevalence of anxiety in patients diagnosed with breast cancer. The Anxiety Sensitivity Index-3 (ASI-3) has been widely used in both general and clinical samples for measuring AS. However, the data about its psychometric properties in women with breast cancer are deficient. In addition, there is no evidence proving the measure equivalence of ASI-3 across sociodemographic variables in the specific sample. Thus, the present study examined the psychometric properties and conducted measure equivalence testing of ASI-3 in Chinese women diagnosed with breast cancer.

**Methods:**

This study included 815 Chinese women diagnosed with breast cancer. Single group confirmatory factor analysis (CFA) was administrated to detect the factorial validity of ASI-3, and multigroup CFAs were conducted to test the measurement equivalence of ASI-3 across various sociodemographic variables. The reliability of ASI-3 was tested by Cronbach’s alpha coefficient, Guttman split-half coefficient, McDonald’s omega coefficient, and test–retest coefficient. The standardized factor loadings, construct reliability, and the average variance extracted of factors were used to measure the construct validity of ASI-3, and the partial correlations were conducted to examine the criterion-related validity of ASI-3.

**Results:**

The ASI-3 had satisfactory reliability and validity in Chinese women diagnosed with breast cancer; three-factor model fitted the data well in CFA and reached partial strict invariances across age, education, and residence groups.

**Conclusion:**

This study explored the psychometric properties and measurement invariance across sociodemographic variables of ASI-3 in Chinese women diagnosed with breast cancer. Our results not only proved that the ASI-3 is an appropriate instrument for measuring AS but also deepened the understanding of ASI-3 in Chinese women with malignancy.

## Introduction

Nowadays, breast cancer is the most common cancer and becomes a leading cause of cancer-related death in Chinese women (Fan et al., 2014). People often experience varying levels of anxiety after the diagnosis of breast cancer, which can bring about harmful effects on their mental health (Burgess et al., 2005). Many studies have proved that anxiety severity was correlated with the levels of anxiety sensitivity (AS) and AS is a trait-like predisposing factor of anxiety (Olatunji and Wolitzky-Taylor, 2009; Knapp et al., 2016; Mohammadkhani et al., 2016). AS refers to individuals’ fear of anxiety-related sensory arousal from the beliefs that the sensation have adverse effects on their body, cognition, and social evaluation (Taylor et al., 2007). Individuals may experience more severe anxiety symptoms by misinterpreting their physical sensations as danger signals (Olatunji and Wolitzky-Taylor, 2009). For example, there is evidence suggesting that women with breast cancer showed more health anxiety, which is strongly associated with the cognitive AS (Jones et al., 2014). Ren et al. (2018) found that neuroticism and AS can positively predict insomnia in women with breast cancer. In general, AS is a specific vulnerability trait for the prevalence of anxiety in patients diagnosed with breast cancer.

The most common instrument for measuring AS is the Anxiety Sensitivity Index-3 (ASI-3), which was established by Taylor et al. (2007) and was proved to have adequate reliability, validity, as well as stable three-factor structure in the United States and Canadian non-clinical samples. At present, ASI-3 has been widely used in different countries. For instance, Ghisi et al. (2016) verified that the ASI-3 was a reliable and valid scale to evaluate AS in the Italian community sample; Kemper et al. (2012) demonstrated that the ASI-3 had a high degree of internal consistency and construct validity in German clinical samples. In addition to these, ASI-3 has been evidenced to have excellent psychometric properties among other countries such as Korean, China, Turkish, and so on (Mantar et al., 2010; Lim and Kim, 2012; Cai et al., 2018). Besides, ASI-3 was also widely applied in various clinical populations such as psychotic disorders, migraine, and cranial meningiomas (Rifkin et al., 2015; Farris et al., 2019; Wagner A. et al., 2019). In terms of factor structure, most previous studies have supported that ASI-3 has a three-factor structure, named physical (six items), social (six items), and cognitive concerns (six items), respectively (Taylor et al., 2007; Kemper et al., 2012; Lim and Kim, 2012; Cai et al., 2018). The physical concerns are positively correlated with body vigilance, social concerns are positively correlated with fear of negative evaluation, and cognitive concerns are most strongly correlated with depression symptoms (Kemper et al., 2012). All in all, the ASI-3 is a satisfactory instrument to evaluate AS in various samples.

Anxiety sensitivity is a trait-like cognitive vulnerability for elevating anxiety levels in patients diagnosed with breast cancer (Olatunji and Wolitzky-Taylor, 2009). However, studies about the application of the ASI-3 in patients with breast cancer are deficient. To explore that whether ASI-3 is also an appropriate tool for measuring AS in patients diagnosed with breast cancer, one aim of the present study was to examine the factor structure, reliability, and validity of ASI-3 in a sample of Chinese women diagnosed with breast cancer. Beyond these, according to previous research, patients with breast cancer in different demographic groups were found to score differently on anxiety-related scales. For example, in Hassan et al.’s (2015) study, the younger age group had a higher risk of anxiety, Wagner J. F. et al. (2019) found patients with young age and low education level performed higher anxiety level. Long-term residence was found to have a significant effect on anxiety in a Chinese sample with breast cancer (Li et al., 2016). More work is needed to explore whether there are differences in vulnerability factors such as AS across various sociodemographic groups in patients with breast cancer. People have studied the invariance of measurement properties of ASI-3 across sex, sexual minority status, age, and race/ethnicity in university students (Jardin et al., 2018). To our knowledge, there is no study proving the measurement invariance of ASI-3 across different demographic groups in patients with breast cancer. It is meaningless to conduct cross-groups comparison of mean differences if the measurement invariance is not established (Schmitt and Kuljanin, 2008). Hence, in current study, we also examined the invariance of factor structure and different performance on ASI-3 across different demographic variables in Chinese women diagnosed with breast cancer.

## Materials and Methods

### Participants

We recruited 1,131 female patients diagnosed with breast cancer at two hospitals in Changsha from March 2011 to March 2016. According to the inclusion criteria, e.g., (1) 20–75 years old, (2) been diagnosed with breast cancer pathologically, (3) are able to read Chinese, (4) were voluntary and completed written informed consent, and (5) no history of major psychiatric disorders or substance abuse, there are 986 qualified candidates. Subsequently, we removed 171 subjects whose questionnaires were incomplete. Finally, this study included 815 patients.

### Measures

#### Anxiety Sensitivity Index-3

It is an 18-item self-report tool for assessing anxiety-related physical (items 3, 4, 7, 8, 12, and 15), cognitive (items 2, 5, 10, 14, 16, and 18), or social (items 1, 6, 9, 11, 13, and 17) concerns. It is a 5-point Likert scale. Each item is scored from 0 to 4, with 0 meaning “I agree very little” and 4 indicating “I agree very much.” Total score is the sum of the score on all items, ranging from 0 to 72. The higher scores on the scale present more severe AS level (Cai et al., 2018).

#### Hamilton Rating Scale for Anxiety

Hamilton et al. developed this questionnaire to assess the anxiety level, which includes 14 items. Half of the items are aimed to test psychic anxiety, and the remaining items measure somatic anxiety. The values on each item range from 0 to 4 (0 = mild anxiety, 2 = moderate anxiety, 3 = severe anxiety, and 4 = very severe or grossly disabling anxiety), and the total scores range from 0 to 56 (Garalejić et al., 2010). The Hamilton Rating Scale for Anxiety (HAMA) is a reliable and valid instrument for measuring anxiety symptoms and widely used in China (Wang C. et al., 2011; Zimmerman et al., 2017).

#### Center for Epidemiologic Studies Depression Scale

The Chinese version Center for Epidemiologic Studies Depression Scale (CESD) is a reliable and effective instrument to assess individuals’ depressive symptoms, including 20 items. Each item is rated from 0 (rarely or none of the time) to 3 (most or all of the time); total scores range from 0 to 60. Higher CESD scores indicated more severe depressive symptoms. In addition, the CESD has a three-factor structure, which includes positive affect, interpersonal problems, as well as depressive mood and somatic symptoms (Zhang et al., 2012).

### Statistical Analysis

SPSS version 22.0 and Mplus version 7.0 were used in this study. Single-group confirmatory factor analysis (CFA) was administrated to verify the three-factor model of ASI-3. Owing to that fact the chi-square (χ^2^) test is sensitive to the sample size, the goodness-of-fit was assessed by the comparative fit index (CFI), Tucker–Lewis index (TLI), root-mean-square error of approximation (RMSEA), and standardized root mean square residual (SRMR). CFI and TLI > 0.90, RMSEA and SRMR < 0.08 indicate an acceptable model fit (Sun, 2005).

Multigroup CFAs were conducted to test the measurement equivalence of ASI-3 across age (younger and older), levels of education (lower and higher), and places of residence (rural and urban). We assessed configural, metric, scalar, and strict invariances across groups in succession, which demonstrated that the composition of latent variables, factor loading of each item, intercepts of the observed variables, and latent variable variation are equal between groups, respectively (Schmitt and Kuljanin, 2008). Given that the χ^2^ change test is also sensitive to the sample size, the results mainly consider the value of the difference of CFI (ΔCFI). According to Cheung and Rensvold (2002) the equivalent model is considered acceptable when ΔCFI < 0.010. As all items obtained significant skewness and kurtosis values (*P* < 0.001) in the Kolmogorov–Smirnov normality test, which means that the data did not fit the normal distribution, so the robust maximum likelihood with mean and variance adjustments estimator was used in all CFAs (Muthén and Muthén, 2012).

The Cronbach’s alpha coefficient, Guttman split-half coefficient, and McDonald’s omega coefficient were used to test the internal consistency of the ASI-3 (Guttman, 1945; Cronbach, 1951; McDonald, 1985). To understand the test–retest reliability of the ASI-3, we randomly recruited 24 patients to complete the ASI-3 again in 2 weeks later, which can test the stability of the ASI-3 across time. Moreover, Mann–Whitney *U*-tests were used to compare the mean differences of ASI-3 scores between age, education, and residence groups.

In terms of construct validity, we tested the criterion-related validity, convergent validity, and discriminant validity of the ASI-3. Partial correlations were conducted to assess the criterion-related validity of ASI-3 against the HAMA and CESD when controlling the effect of age. In addition, the Harman’s single factor test was performed to examine the effects caused by common method variance. The hypothesis is that if the covariation could largely be explained by method variance, a singer factor (method factor) should exist in the variables of these measures (Harris and Mossholder, 1996). According to Hair et al. (2010), the convergent validity could be measured by standardized factor loadings, construct reliability (CR), and average variance extracted (AVE) of factors. If the standardized factor loadings of ASI-3 are >0.5 and statistically significant, the value of CR and AVE of each factor is higher than 0.7 and 0.5, respectively, the ASI-3 has satisfied convergent validity. Discriminant validity was examined by comparing the AVE with the square of the latent correlations among factors. When the AVE of the ASI-3 is above the square of the latent correlations, the ASI-3 has an acceptable discriminant validity (Hair et al., 2010).

## Results

### Descriptive Statistics

A sample of 815 female patients diagnosed with breast cancer from two hospitals in Changsha participated in this study. These participants aged 26–71 years (mean age = 47.66 years, SD = 8.35). The specific information is summarized in Tables 1, 2.

**TABLE 1:** Demographic and disease-related information of the patients.

**Item**	***N* (%)**
Years of age	
≤50	555 (68.1%)
>50	260 (31.9%)
Marital status	
Married	765 (93.8%)
Single	3 (0.4%)
Divorced	29 (3.6%)
Widowed	18 (2.2%)
Educational level	
≤6 years	148 (18.2%)
>6 years	667 (81.8%)
Places of residence	
Urban	409 (50.2%)
Rural	406 (49.8%)
Period	
Preoperative	235 (28.8%)
Postoperative	550 (67.5%)
Convalescent	30 (3.7%)

**TABLE 2:** The descriptive statistics and the reliability parameters of the instruments used in this study.

**Instruments**	***M***	**SD**	**Skewness**	**Kurtosis**	**Cronbach’s alpha**	**McDonald’s omega**
					**coefficients (90% CI)**	**coefficient (90% CI)**
ASI-3 (*N* = 815)	13.659	11.766	1.075	0.779		
Physical concerns	3.993	4.488	0.990	0.082	0.936 (0.930–0.942)	0.902 (0.890–0.914)
Social concerns	5.371	5.221	0.960	0.378	0.924 (0.917–0.930)	0.885 (0.871–0.899)
Cognitive concerns	4.296	4.474	1.228	1.253	0.935 (0.929–0.941)	0.898 (0.885–0.911)
HAMA (*N* = 604)	10.320	5.271	0.170	–0.227	0.774 (0.751–0.796)	0.733 (0.715–0.752)
CESD (*N* = 428)	19.923	10.099	–0.051	–1.096	0.683 (0.646–0.719)	0.783 (0.766–0.799)

*ASI-3, Anxiety Sensitivity Index-3; HAMA, Hamilton Anxiety Scale; CESD, Center for Epidemiologic Studies Depression Scale.*

### Confirmatory Factor Analysis

Confirmatory factor analysis was administrated to examine the three-factor model of ASI-3 in this study. The fit indices were as follows: χ^2^ = 474.881, *df* = 132, CFI = 0.930, TLI = 0.919, RMSEA = 0.056 (90% confidence interval = 0.051–0.062), and SRMR = 0.046. All indices meet the fitting requirements, demonstrating that the three-factor model had adequate factorial validity in current sample. The standardized factor loadings obtained in CFA and the latent correlations among factors are presented in Table 3.

**TABLE 3:** Item loadings of three-factor structure and latent correlations among factors in CFA.

		**Standardized factor loadings**		
		**Factor I**	**Factor II**	**Factor III**
**Physical concerns**				
Item 3	It scares me when my heart beats rapidly.	0.844		
Item 4	When my stomach is upset, I worry that I might be seriously ill.	0.844		
Item 7	When my chest feels tight, I get scared that I won’t be able to breathe properly.	0.885		
Item 8	When I feel pain in my chest, I worry that I’m going to have a heart attack.	0.903		
Item 12	When I notice my heart skipping a beat, I worry seriously wrong with me.	0.775		
Item 15	When my throat feels tight, I worry that I could choke to death.	0.816		
**Social concerns**				
Item 1	It is important for me not to appear nervous.		0.740	
Item 6	When I tremble in the presence of others, I fear what people might think of me.		0.816	
Item 9	I worry that other people will notice my anxiety.		0.917	
Item 11	It scares me when I blush in front of people.		0.838	
Item 13	When I begin to sweat in a social situation, I fear people will think negatively of me.		0.870	
Item 17	I think it would be horrible for me to faint in public.		0.750	
**Cognitive concerns**				
Item 2	When I cannot keep my mind on a task, I worry that I might be going crazy.			0.851
Item 5	It scares me when I am unable to keep my mind on a task.			0.883
Item 10	When I feel “spacey” or spaced out, I worry that I may be mentally ill.			0.830
Item 14	When my thoughts seem to speed up, I worry that I might be going crazy.			0.834
Item 16	When I have trouble thinking clearly, I worry there is something wrong with me.			0.846
Item 18	When my mind goes blank, I worry there is something terribly wrong with me.			0.800

		**Latent correlations (90% CI)**		

Physical concerns with social concerns		0.495 (0.440–0.549)		
Physical concerns with cognitive concerns		0.531 (0.482–0.580)		
Social concerns with cognitive concerns		0.658 (0.616–0.700)		

*All of the standardized factor loadings are statistically significant.*

### Measurement Invariance of ASI-3 Three-Factor Model Across Demographic Groups

The fit indices of CFA in demographic subgroups are presented in Table 4. We found that the fitting indices did not totally meet the fitting conditions in older group (>50), lower education group (≤6 years), and urban group. According to the modified index (MI), we selected the largest value of MI in turn to free the parameters. In the older group (>50), there were correlated measurement errors between items 2 and 5 [Cov(*y*2, *y*5)] as well as items 11 and 13 [Cov(*y*11, *y*13)], the error correlation coefficient was 0.595 (*p* < 0.01) for Cov(*y*2, *y*5) and 0.489 (*p* < 0.01) for Cov(*y*11, *y*13); with regard to lower education group (≤6 years), the measurement error between items 10 and 14 was correlated [Cov(*y*10, *y*14)], and the correlation coefficient was 0.611 (*p* < 0.01). Concerning the urban group, we correlated the measurement error of item 2 with item 5, and the error correlation coefficient was 0.510 (*p* < 0.01). The three-factor model of ASI-3 had adequate fit among all subgroups after freed above parameters which are also summarized in Table 4. Actually, any parameter to be released must be highly consistent with the theory (MacCallum et al., 1992). We found that the description of item 2 (When I cannot keep my mind on a task, I worry that I might be going crazy) and item 5 (It scares me when I am unable to keep my mind on a task) are similar; meanwhile, they both load on cognitive concerns dimension; as for item 11 (It scares me when I blush in front of people) and item 13 (When I begin to sweat in a social situation, I fear people will think negatively of me), both of them express individual’s worry about somatic symptoms in front of others and belong to the same factor; item 10 (When I feel “spacey” or spaced out, I worry that I may be mentally ill) and item 14 (When my thoughts seem to speed up, I worry that I might be going crazy) express the concerns about the instability of cognitive state and belong to cognitive concerns.

**TABLE 4:** Goodness-of-fit indices of confirmatory factor analysis (CFA) in demographic subgroups.

**Item**		**χ^2^**	***df***	**CFI**	**TLI**	**RMSEA (90% CI)**	**SRMR**
Years of age	≤50	393.432	132	0.917	0.903	0.060 (0.053–0.067)	0.048
	>50	261.747	132	0.897	0.880	0.061 (0.051–0.072)	0.066
Modification	>50*^$^	223.255	130	0.926	0.912	0.053 (0.041–0.064)	0.062
Educational level	≤6 years	210.817	132	0.897	0.881	0.064 (0.047–0.079)	0.048
	>6 years	413.639	132	0.920	0.908	0.057 (0.050–0.063)	0.051
Modification	≤6 years^#^	197.065	131	0.914	0.900	0.058 (0.041–0.075)	0.047
Places of residence	Urban	332.046	132	0.903	0.887	0.061 (0.053–0.069)	0.057
	Rural	326.636	132	0.924	0.912	0.057(0.049–0.065)	0.056
Modification	Urban*	304.219	131	0.916	0.901	0.057 (0.049–0.065)	0.053

*Items 2 and 5 correlated. ^$^Items 11 and 13 correlated. ^#^Items 10 and 14 correlated. CFI, comparative fit index; TLI, Tucker–Lewis index; RMSEA, root-mean-square error of approximation; SRMR, standardized root mean square residual.

According to Byrne et al. (1989), baseline models are not required to be identical across groups in invariance testing; therefore, we established the multigroup model based on different baseline models in measurement invariance testing.

First, we examined the measurement invariance across different age groups (≤50 years old vs. >50 years old). In the multigroup model, there were Cov(*y*2, *y*5) and Cov(*y*11, *y*13) in the older group (>50), and there is no modification in the younger group (≤50). We test the configural, metric, scalar, and strict invariance between two groups. The ΔCFI between the configural invariance model and metric invariance model was 0.001, ΔCFI between the metric invariance model and scalar invariance model was 0.003, and ΔCFI between the scalar invariance model and strict invariance model was 0.002. All of them were below than 0.01.

Second, we evaluated the equivalence of the factor structure between two groups with lower (≤6 years) and higher (>6 years) educational level, respectively. In the multigroup model, there was just a Cov(*y*10, *y*14) in the lower education group (≤6 years). All of the ΔCFIs were 0.002 when compared the constrained model with the unconstrained model.

Finally, we tested invariance between urban and rural groups. In the multigroup model, there was a Cov(*y*2, *y*5) only in the urban group. We found that all invariance models fit the data well. The differences in CFI between the unconstrained model and the constrained model were 0.004 (configural invariance vs. metric invariance), 0.003 (metric invariance vs. scalar invariance), and 0.004 (scalar invariance vs. strict invariance). Table 5 summarized the goodness-of-fit statistics of measurement equivalence analysis.

**TABLE 5:** Measurement invariance testing of the Anxiety Sensitivity Index-3 (ASI-3).

**Item**	**χ^2^**	***df***	**CFI**	**TLI**	**RMSEA (90% CI)**	**ΔCFI**
**Years of age (≤50 vs. >50)**						
Configural invariance	591.776	262	0.919	0.906	0.058 (0.050–0.062)	
Metric invariance	612.192	277	0.918	0.909	0.054 (0.049–0.060)	0.001
Scalar invariance	639.807	292	0.915	0.911	0.054 (0.048–0.060)	0.003
Strict invariance	648.743	310	0.917	0.918	0.052(0.046–0.057)	0.002
**Educational level (≤6 vs. >6 years)**						
Configural invariance	577.834	263	0.920	0.906	0.054 (0.048–0.060)	
Metric invariance	597.364	278	0.918	0.910	0.053 (0.047–0.059)	0.002
Scalar invariance	622.582	293	0.916	0.912	0.053 (0.047–0.058)	0.002
Strict invariance	633.086	311	0.918	0.919	0.050(0.045–0.056)	0.002
**Places of residence (urban vs. rural)**						
Configural invariance	598.601	263	0.922	0.909	0.056 (0.050–0.062)	
Metric invariance	631.396	278	0.918	0.910	0.056 (0.050–0.062)	0.004
Scalar invariance	660.025	293	0.915	0.911	0.055 (0.050–0.061)	0.003
Strict invariance	694.632	311	0.911	0.912	0.055(0.050–0.061)	0.004

*CFI, comparative fit index; TLI, Tucker–Lewis index; RMSEA, root-mean-square error of approximation; ΔCFI, change in comparative fit index.*

In summary, given the modifications in the multigroup models, there were partial strict invariances across age, education, and residence groups.

### Reliability

In our study, ASI-3 was proved to have a three-factor structure. Therefore, we examined the reliability coefficient for each factor of the ASI-3. The statistics of Cronbach’s alpha coefficients (0.924–0.936) and McDonald’s omega coefficients (0.885–0.902) are summarized in Table 2, and the Guttman split-half coefficient of the physical concerns, social concerns, and cognitive concerns was 0.938, 0.906, and 0.918, respectively, suggesting that the three factors of ASI-3 had satisfactory reliability. The test–retest coefficient for the physical concerns, social concerns, and cognitive concerns was 0.775, 0.596, and 0.730, respectively (*P* < 0.01), which revealed a moderate correlation between test and retest results (Lee et al., 2018). Moreover, we summarized the reliability parameters of other instruments used in our study in Table 2.

### Mann–Whitney *U*-Tests

The specific information is shown in Table 6. We found that there was just a significant difference in cognitive concerns between age groups (*P* < 0.01). Lower education group had significantly higher scores on ASI-3 compared with higher education group except for cognitive concerns dimension (*P* < 0.01). The ASI-3 scores in rural group were significantly higher than urban group (*P* < 0.05). However, the effect sizes of all significant differences were lower than 0.3 (Fritz et al., 2012).

**TABLE 6:** Comparison of ASI-3 scores across groups.

**Variables**			**Mean difference**	***P***	***r***
Years of age	≤50	>50			
ASI-3	13.85 ± 11.81	13.26 ± 11.67	0.59	0.378	
Physical concerns	3.91 ± 4.21	4.17 ± 4.63	0.26	0.622	
Cognitive concerns	4.57 ± 4.56	3.71 ± 4.24	0.86	0.004	0.102
Social concerns	5.37 ± 5.10	5.38 ± 5.47	0.01	0.622	
Educational level	≤6 years	>6 years			
ASI-3	17.10 ± 13.84	12.90 ± 11.12	4.20	0.002	0.106
Physical concerns	5.39 ± 4.76	3.68 ± 4.37	1.71	0.000	0.142
Cognitive concerns	5.05 ± 5.18	4.13 ± 4.29	0.92	0.102	
Social concerns	6.66 ± 5.89	5.09 ± 5.02	1.57	0.008	0.094
Places of residence	Urban	Rural			
ASI-3	12.55 ± 11.04	14.78 ± 12.37	2.23	0.013	0.087
Physical concerns	3.70 ± 4.46	4.29 ± 4.50	0.59	0.018	0.083
Cognitive concerns	3.95 ± 4.25	4.64 ± 4.67	0.69	0.027	0.077
Social concerns	4.89 ± 4.96	5.85 ± 5.43	0.96	0.011	0.089

*ASI-3, Anxiety Sensitivity Index-3.*

### Construct Validity

Harman’s single factor test revealed that the one-factor structure did not fit the data well, and the fit indices were as follows: χ^2^ = 11,715.484, *df* = 1,274, CFI = 0.296, TLI = 0.268, RMSEA = 0.139 (90% confidence interval = 0.137–0.141), and SRMR = 0.151. Therefore, the common method variance did not have a serious effect on our following results.

Partial correlations were conducted to test the criterion-related validity of ASI-3 when controlling for the effect of age and shown in Table 7. We found that the scores of the three factors were highly and positively interrelated with total score (*r* = 0.777–0.867, *P* < 0.01) and moderately correlated with each other (*r* = 0.488–0.581, *P* < 0.01). The significant correlation between ASI-3 total score and HAMA (*r* = 0.505, *P* < 0.01) revealed that the ASI-3 had an acceptable empirical validation. Furthermore, the cognitive concerns dimension had greater correlations with the total score (*r* = 0.394, *P* < 0.01) and two factors (positive affect, *r* = −0.242, *P* < 0.01; depressive mood and somatic symptoms, *r* = 0.436, *P* < 0.01) of CESD comparing to other factors and total scale of ASI-3, while the social concerns had a greatest correlation with the interpersonal problems dimension of CESD (*r* = 0.222, *P* < 0.01). In addition, the standardized factor loadings for all items in ASI-3 ranged from 0.740 to 0.917 and statistically significant, which are presented in Table 3. The value of AVE of each factor ranged from 0.678 to 0.715, and the value of CR ranged from 0.926 to 0.938, which are summarized in Table 8. The standardized factor loadings, AVE of factors, and CR were united to suggest that the ASI-3 had strong convergent validity. In addition, the values of AVE of three factors were higher than the square of the latent correlations among factors (ranged from 0.245 to 0.433), indicating a satisfied discriminant validity of ASI-3.

**TABLE 7:** Partial correlations among ASI-3 and other measures.

	**ASI-3**	**Physical**	**Cognitive**	**Social**
		**concerns**	**concerns**	**concerns**
Physical concerns	0.777**			
Cognitive concerns	0.835**	0.488**		
Social concerns	0.867**	0.513**	0.581**	
HAMA	0.505**	0.294**	0.492**	0.450**
CESD	0.318**	0.130**	0.394**	0.252**
CESD – positive affect	−0.189**	−0.054	−0.242**	−0.159**
CESD – interpersonal problems	0.187**	0.117*	0.110*	0.222**
CESD – depressive mood and somatic symptoms	0.338**	0.129**	0.436**	0.256**

The effects of age were included in the analysis. ASI-3, Anxiety Sensitivity Index-3; HAMA, Hamilton Anxiety Scale; CESD, Center for Epidemiologic Studies Depression Scale. *P < 0.05. **P < 0.01.

**TABLE 8:** The value of construct reliability (CR) and the average variance extracted (AVE) for each factor in ASI-3.

**Factor**	**CR**	**AVE**
Physical concerns	0.938	0.715
Social concerns	0.926	0.678
Cognitive concerns	0.935	0.707

*CR, construct reliability; AVE, average variance extracted.*

## Discussion

Anxiety Sensitivity Index-3 is a widely used instrument for measuring AS. This study is aimed at exploring the psychometric properties of ASI-3 in Chinese women with breast cancer. Many previous studies have proven that the ASI-3 had a three-factor structure when applied in different cultural backgrounds and populations (Taylor et al., 2007; Mantar et al., 2010; Lim and Kim, 2012; Ghisi et al., 2016). To verify the factor validity of ASI-3, we conducted CFA in current sample. The satisfactory fitting indices illustrated that the three-factor model was practicable in Chinese women with breast cancer. The standardized factor loadings and the latent correlations among factors testified that the three factors are not only relative independent but also correlated with each other.

Measurement equivalence is the premise for comparing the scores on ASI-3 across different groups (Schmitt and Kuljanin, 2008); therefore, we conducted multigroup CFA to test the measurement invariance before compared the scores on ASI-3 across various sociodemographic groups. We found that the baseline models were different across groups when analyzing the three-factor structure separately for each group. Specifically, there were Cov(*y*2, *y*5) and Cov(*y*11, *y*13) in the older group (>50), Cov(*y*10, *y*14) in the lower education group (≤6 years), and Cov(*y*2, *y*5) in the urban group. All of the modifications were based on the MI applied by Mplus and sufficient theoretical meaning because the three pairs of correlated items have similar directionality and load on the same factor in our study. Particularly, for the older group and lower education group, it might be difficult for them to distinguish the related items due to their relatively lower faculty of understanding. Moreover, the sample size could affect the analysis of CFA and model modifications (MacCallum et al., 1992). In terms of the modification in the urban group, generally, urban residents are more engaged in mental activities, while rural residents are more engaged in manual labor; thus, when people cannot keep focus on their work, urban residents are more likely to ascribe the reason to cognitive problems, while rural residents are more likely to attribute the reason to tired body. It may be the reason why we correlated measurement errors between items 2 and 5 in urban group but not in rural group. All in all, baseline models are not required to be identical across groups (Byrne et al., 1989; Wang J. et al., 2011). Therefore, we carried out measurement equivalence testing based on these results and found the model of three-factor structure reached partial strict invariances across demographic groups. In addition, Cronbach’s alpha coefficients, McDonald’s omega coefficients, and Guttman split-half coefficients of the three factors of ASI-3 all indicated that the ASI-3 was a reliable instrument to test the three aspects – physical, social, and cognitive concerns of AS in Chinese women with breast cancer. The test–retest coefficient revealed that the three factors of ASI-3 remained relatively stable over a 2-week period.

Subsequently, the results of Mann–Whitney *U* tests revealed that the score on cognitive concerns was significantly higher in younger group than in older group. Previous studies have shown that breast cancer patients with younger age showed higher levels of anxiety (Jones et al., 2014; Hassan et al., 2015; Wagner J. F. et al., 2019). AS is a trait-like cognitive vulnerability of anxiety; therefore, it is understandable to get a higher score of the ASI-3 for younger patients. However, the differences were not observed in physical and social concerns. In addition, the ASI-3 scores were significantly higher in the lower education group than in the higher education group, except for cognitive concerns dimension, and the ASI-3 scores were significantly higher in the rural group compared with the urban group. Actually, for patients with breast cancer, long-term residence and education level have an impact on their cognitive range and ability (Li et al., 2016). Relatively speaking, patients in rural and with lower education might have more fragile and sensitive perception, which may be the reason for the higher scores of the ASI-3 in the rural group and in the lower education group. Notably, the effect sizes of these significant differences were small, which indicated that the results might be overvalued because of the large sample size (Fritz et al., 2012; Tomczak and Tomczak, 2014).

The moderate correlation between ASI-3 and the measures of anxiety indicated that the ASI-3 had an adequate empirical validation. In the meantime, the cognitive concerns were positively and moderately related with the depressive mood and somatic symptoms as well as the total score of CESD, which manifested that the cognitive concerns were uniquely relevant to depressive symptoms; it is consistent with the findings of previous research (Olthuis et al., 2014). Moreover, the social concerns performed a greatest correlation with the interpersonal problems, which was accordant with our hypothesis. Moreover, either the standardized factor loadings or the value of AVE and CR were well above the threshold suggested by Hair et al. (2010), indicating that the variances were more explained by each factor and all of the items of each factor were consistent for measuring the same latent construct. Meanwhile, the AVE of any factor was higher than the square of the latent correlations among the factors, which indicated that the three factors could extract more variance than the sharing among factors. To sum up, the ASI-3 had satisfied convergent and discriminant validity in Chinese women diagnosed with breast cancer (Hair et al., 2010).

There were several limitations in this study. First, there are other contents about criterion-related validity of ASI-3 that were not explored; thus, more researches are needed to complement relevant information. Second, we just roughly divided the age and educational level into two groups; the contents about the measurement equivalence across more age and education groups need further supplement. Third, we only collected 24 patients in the test–retest reliability analysis; the number of cases is relatively small, so the cross-time stability of the factor structure needs to be further confirmed by future studies. Finally, the social desirability problems might have an effect on our results, and more researches are needed to explore the degree of the effects.

In summary, we explored the factor structure and psychometric properties of the Chinese version ASI-3 in female patients with breast cancer. In spite of the limitations mentioned above, we have expanded the use of ASI-3 in Chinese women diagnosed with breast cancer. Our findings not only demonstrated that the ASI-3 had adequate reliability, validity, and a stable three-facture structure but also initially approved its strict sociodemographic measurement equivalence.

## Data Availability Statement

The datasets generated for this study are available on request to the corresponding author.

## Ethics Statement

The studies involving human participants were reviewed and approved by the Ethics Committee of the Second Xiangya Hospital, Central South University. The patients/participants provided their written informed consent to participate in this study.

## Author Contributions

YH, LL, XL, and XZ conceived and designed the study. JZ collected the data and revised the manuscript. YH, HZ, and SL organized and supervised the data collection and inputting. YH drafted the manuscript and organized and supervised the data analysis. JF, JZ, and YY provided the critical comments on various drafts of the manuscript. All authors read and approved the final manuscript.

## Conflict of Interest

The authors declare that the research was conducted in the absence of any commercial or financial relationships that could be construed as a potential conflict of interest.
